# Improving Efficacy of *Beauveria bassiana* against Stored Grain Beetles with a Synergistic Co-Formulant

**DOI:** 10.3390/insects7030042

**Published:** 2016-08-26

**Authors:** Clare Storm, Freya Scoates, Adam Nunn, Olivier Potin, Aoife Dillon

**Affiliations:** 1 Exosect Ltd., Leylands Business Park, Colden Common, Winchester SO21 1TH, UK; freya.scoates@exosect.com (F.S.); adam.nunn@myport.ac.uk (A.N.); aoife.dillon@exosect.com (A.D.); 2 Agrauxine, 18 Route De Mauvieres, Loches 37600, France; olivier.potin@agrauxine.fr

**Keywords:** co-formulant, biopesticide, synergy, *Beauveria bassiana*, kaolin, entomopathogenic fungi

## Abstract

The potential of a dry powder co-formulant, kaolin, to improve the control of storage beetles by the entomopathogenic fungus *Beauveria bassiana*, isolate IMI389521, was investigated. The response of *Oryzaephilus surinamensis* adults to the fungus when applied to wheat at 1 × 10^10^ conidia per kg with and without kaolin at 1.74 g per kg wheat was assessed. Addition of kaolin increased control from 46% to 88% at day 7 and from 81% to 99% at day 14 post-treatment. Following this the dose response of *O. surinamensis* and *Tribolium confusum* to both kaolin and the fungus was investigated. Synergistic effects were evident against *O. surinamensis* at ≥0.96 g of kaolin per kg of wheat when combined with the fungus at all concentrations tested. For *T. confusum*, adult mortality did not exceed 55%, however, the larvae were extremely susceptible with almost complete suppression of adult emergence at the lowest fungal rate tested even without the addition of kaolin. Finally, the dose response of *Sitophilus granarius* to the fungus at 15 and 25 °C, with and without kaolin at 1 g per kg of wheat, was examined. Improvements in efficacy were achieved by including kaolin at every fungal rate tested and by increasing the temperature. Kaolin by itself was not effective, only when combined with the fungus was an effect observed, indicating that kaolin was having a synergistic effect on the fungus.

## 1. Introduction

Stored-product insects and mites cause serious post-harvest losses, estimated to range from 9% in developed countries to 20% or more in developing countries [1]. Pest infestations reduce the value of the commodity by contaminating it with insect fragments, faeces, webbing, and metabolic by-products [2]. The majority of grain stores and exporters have zero tolerance for pests and pesticide residues. Concerns over insecticide resistance, residues, and environmental impacts together with changes in legislation have led to a decline in available pesticides to protect stored food. Alternative pest control products are required to maintain levels of food production and anticipate future demand.

The use of entomopathogenic fungi such as *Beauveria bassiana* (Balsamo) Vuillemin (Hypocreales: Cordycipitaceae) and *Metarhizium anisopliae* (Metschinkoff) Sorokin (Hypocreales: Clavicipitaceae) to control stored product insects and mites has been explored extensively in laboratory and field scale trials [3,4,5,6,7,8]. Despite positive results, no microbial control agents based on entomopathogenic fungi are currently commercially available for grain storage. Perceptions that such products deliver lower efficacy than conventional chemical control measures may be one barrier to their commercialisation. These perceptions may be counteracted by improving the formulation and delivery of microorganisms.

Inert dusts, such as mineral clays and silica powders, kill arthropods by removing the epicuticular lipid layers causing excessive water loss through the cuticle [9,10]. These dusts have been widely used for stored product pest control around the globe [11,12,13]. Of the inert dusts Diatomaceous earth (DE), a silica-based sedimentary rock, is the most widely used for storage protection and has been used commercially as a standalone preventative and curative treatment against stored product pests. DE demonstrates efficacy against a wide range of pests [14,15,16,17], but its widespread adoption has been hampered based on a number of perceived drawbacks: (1) its effectiveness varies widely depending on target species and life stage, humidity, and temperature [14,15,18], (2) problems with processing machinery maintenance & damage [10], and (3) reductions in grain quality parameters such as bulk density and flowability [19,20]. Kaolin, an alternative inert dust, is a common, silica-based clay mineral used in industrial manufacturing [21]. It is a siliceous mineral similar to, but softer than, DE; it scores relatively low (2.0–2.5) on Mohs hardness index, making it less likely to abrade milling equipment (Campden BRI, pers. comm). Kaolin has demonstrated effectiveness on stored product insects [22,23] but requires very high application rates in admixture with grain (5–10 g per kg of grain) when used as a standalone product [10,24]. Such inclusion rates would have an unacceptable impact on grain quality parameters such as appearance, bulk density and flowability. However, as with DE, there may be the potential to reduce application rates if it can be coupled with another active ingredient.

Several studies have demonstrated that inert dusts can be used as a carrier of microbial control agents in stored product protection. In the case of DE additive [25,26,27,28], and sometimes synergistic effects [6,29,30], have been observed. Lord (2001) [29] found that when *B. bassiana* was co-formulated with DE a synergistic effect was observed against the sawtoothed grain beetle, *Oryzaephilus surinamensis* (L.) (Coleoptera: Silvanidae), the lesser grain borer, *Rhyzopertha dominica* (F.) (Coleoptera: Bostrichidae) and the rusty grain beetle, *Cryptolestes ferrugineus* (Stephens) (Coleoptera: Laemophloeidae). Akbar et al. (2004) [30] also recorded a synergistic effect when testing the same combination against *Tribolium castaneum* (Herbst) (Coleoptera: Tenebrionidae) larvae. Sabbour et al., (2012) [6] tested *M. anisopliae* and *B. bassiana* isolates against storage moths *Plodia interpunctella* Hübner (Lepidoptera: Pyralidae), *Ephestia cautella* Walker (Lepidoptera: Pyralidae), and *Ephestia kuehniella* Zeller (Lepidoptera: Pyralidae) and found that, in most cases, DE had a synergistic effect when combined with the fungi. Improvements in efficacy against storage pests have also been reported when *M. anisopliae* [26,27] and *Paecilomyces fumosoroseus* (Wise) Brown and Smith (Eurotiales: Trichocomaceae) [28] were combined with DE, compared to when the fungus was applied alone. There are fewer examples where the combination of an entomopathogenic fungus with the inert dust kaolin has been tested on storage pests. Samodra & Ibrahim (2006a, b) [31,32] found that *B. bassiana* formulated in kaolin was more efficacious against larvae of the rice moth, *Corcyra cephalonica* Stainton (Lepidoptera: Pyralidae) and adult *Sitophilus oryzae* (L.) (Coleoptera: Curculionidae), than when the fungus was formulated in tapioca flour or unformulated.

In this study, we evaluated the ability of kaolin to enhance the activity of *B. bassiana*, isolate IMI389521, against stored grain beetles in a series of bioassays. Our hypothesis was that the presence of kaolin would increase the pathogenicity of the fungus to the target insects. A variety of adult beetles were tested to determine if any effect of kaolin was species-dependent. As test insects we used three major stored product beetle species with varying susceptibility to the fungus: the saw-toothed grain beetle *O*. *surinamensis*, the granary weevil *S. granarius*, and the relatively tolerant [32] confused flour beetle *Tribolium confusum* Jacquelin du Val (Coleoptera: Tenebrionidae). *Sitophilus granarius* is a primary pest, infesting intact kernels, while *T. confusum* and *O. surinamensis* are secondary pests, infesting only damaged or broken kernels [33] and processed grain such as flour. Additionally, due to the relatively low levels of mortality observed on adult *T. confusum*, the response of the larvae, which have been shown to be more susceptible to entomopathogenic fungi than the adults [26,30], were also tested.

## 2. Materials and Methods

### 2.1. Test Insects

All adult insects were taken from laboratory maintained cultures. The *O. surinamensis* individuals were reared on rolled oats and wheat germ in a ratio of 3:1 *w*/*w* at 27 ± 2 °C and 40% ± 5% relative humidity (RH). The *T. confusum* adults were maintained on a diet of rolled porridge oats and brewer’s yeast in a ratio of 9:1 *w*/*w* at 30 ± 2 °C and 40% ± 5% RH. Adult *S. granarius* were reared on kibbled wheat grain and wheat germ in a ratio of 19:1 *w*/*w* at 25 ± 2 °C and 65% ± 5% RH. All insects were reared in continuous darkness. Adult beetles of mixed age and sex were used for testing.

For the larval bioassay (bioassay 3) all of the early instar *T. confusum* larvae required were supplied in diet from i2L Research (Cardiff, UK).

### 2.2. Test Items

The isolate of *B. bassiana* IMI389521 was originally sourced from an infected adult coleopteran *S. oryzae* in a UK grain store [5] and had previously demonstrated efficacy against a variety of storage insects, good stability in storage, and high levels of viability following mass-production [34,35]. The isolate was manufactured by Agrauxine (Loches, France). The dry conidia were combined with co-formulants at Exosect Ltd. (Winchester, UK) to make the various formulations. In bioassay 1 one the *B. bassiana* quantities are expressed as total conidia per kg of wheat but in subsequent bioassays, due to a change in quality control procedures by the manufacturer, the quantities are expressed as colony forming units (CFU) per kg wheat. Kaolin clay (AgriBind™) was supplied by Imerys (Par, Cornwall, UK). In the *S. granarius* experiment, bioassay 4, the conidia were also combined with Entostat^®^ to aid dispersion and adhesion to grain and insects, and silica to aid flow. The Entostat variant used was micronized carnauba wax supplied by Exosect Ltd. and Sipernat d17 was supplied by Lawrence Industries (Tamworth, UK).

### 2.3. Commodity

Residue-free wheat was supplied from Street End Farm (Bishops Waltham, UK) and kibbled for 30 s in a coffee grinder, except for the *S. granarius* bioassay (bioassay 4), where residue-free wheat grain var. Alderon was supplied by KWS UK (Royston, UK) and was not kibbled.

Grain used in bioassay three and four was stored in paper bags in 500 g samples and placed in a humidity-controlled incubator (TK 252, Nüve, Ankara, Turkey)) for one week before the bioassays in order to equilibrate the grain to the test conditions.

### 2.4. Bioassay 1: Initial Testing of O. surinamensis

The response of *O. surinamensis* adults to *B. bassiana* IMI389521 at 1 × 10^10^ conidia per kg of wheat was tested with and without kaolin admixed at a rate of 1.74 g per kg of wheat. Kaolin without *B. bassiana* and an untreated control were included for comparison. Fifty grams of kibbled wheat were weighed into 125 mL glass jars and treatments were added on top. The jars were covered and placed on a vortex mixer for 10 s to disperse the treatments. Five replicate jars were created for each treatment. Twenty adult *O. surinamensis* were added to each jar and the jars were covered with a piece of gauze held in place with an elastic band. Jars were arranged on a shelf in a bioassay room set to constant darkness (except during mortality checks) and 25 ± 3 °C. Humidity was monitored throughout the trial with a Lascar data logger and found to range from 47% to 62% RH.

At days 7 and 14 the contents of each pot were individually tipped onto a white plastic tray and the insects were checked for mortality. Dead beetles were removed. Live beetles, grain, and powder were tipped back into the pots, except at the final time point. Trays were sterilized with 70% ethanol between treatments.

### 2.5. Bioassay 2: Dose Response of O. surinamensis

The response of *O. surinamensis* adults to different rates of *B. bassiana* IMI389521 and kaolin was tested. The fungus was applied at five rates: 0, 5.4 × 10^8^, 1.7 × 10^9^, 5.4 × 10^9^, and 1.7 × 10^10^ CFU per kg of wheat, and for each rate of fungus the kaolin was tested at one of four rates: 0, 0.096, 0.963, and 1.925 g per kg of wheat, resulting in 20 treatment groups in total.

For each bioassay and treatment group 250 g of kibbled wheat was weighed into a 1 L glass Kilner jar and treatments were added on top. The jars were rolled by hand for 30 s to disperse the treatments. The grain was then subdivided into five replicate 125 mL glass jars. Twenty adult *O. surinamensis* were added to each jar and the jars were covered with a piece of gauze held in place with an elastic band. Jars were arranged in a humidity controlled incubator set to 25 ± 2 °C and 65% ± 5% RH in constant darkness. At day 14, the insects were checked for mortality.

### 2.6. Bioassay 3: Dose Response of T. confusum Adults and Larvae

The response of *T. confusum* adults and larvae to different rates of *B. bassiana* IMI389521 and kaolin was tested. The response of *T. confusum* was assessed at higher rates of *B. bassiana* than of *O. surinamensis*, as the adults are less susceptible to the fungus than the other beetles tested [34]. In addition, lower concentrations of kaolin were assessed in the *T. confusum* bioassays as the highest rate (1.925 g per kg of wheat) was subsequently considered to be not commercially viable. The fungus was applied at five rates: 0, 1.78 × 10^10^, 3.16 × 10^10^, 5.62 × 10^10^, and 1 × 10^11^ CFU per kg of wheat in both bioassays. In the adult bioassay, for each rate of fungus the kaolin was applied at one of five rates: 0, 0.178, 0.316, 0.562, and 1 g per kg of wheat resulting in 25 treatment groups in total. In the larval bioassay, kaolin was tested at either 0 or 0.562 g per kg of wheat, resulting in ten treatment groups in total. 

In the adult bioassay, the grain was treated, sub-divided, and insects added as per Section 2.5. In the larval bioassay, it was not possible to count out larvae for each replicate jar so the container of larvae in diet, with an estimated content of 2500 larvae, was homogenized by gently rolling for 30 s and then subdivided by weight into ten samples, one for each treatment group. The treatments were applied to the grain as per Section 2.5 and then the larvae were added before sub-dividing the mixture into five replicate jars. Assuming a homogenous mixture of larvae in diet, this should have resulted in approximately 250 larvae per treatment group and then 50 larvae per replicate jar. The jars in both bioassays were stored as per Section 2.5.

At day 14, the adult insects were checked for mortality as detailed in Section 2.4. At day 42, the jars from the larval bioassay were checked for adult emergence.

### 2.7. Bioassay 4: Dose Response of S. granarius and the Effect of Temperature

The response of *S. granarius* adults to different rates of *B. bassiana* IMI389521 with and without kaolin at 1 g per kg wheat was assessed. The fungus was applied at four rates: 0, 1.78 × 10^9^, 5.62 × 10^10^, and 1.78 × 10^10^ CFU per kg wheat. For each of the formulations containing the fungus, a flow agent, (Sipernat d17) and Entostat, were also added. Sipernat d17 was added as 0.5% *w*/*w* of the total quantity of formulation to be applied. The balance of the formulation was made up of Entostat, which was added to the dry conidia before mixing with the kaolin. Details of the treatments, application rates and quantities of components are given in Table 1. An untreated control and kaolin-only treatment were also assessed. Mortality was examined 14 and 28 days after incubation at either 15 or 25 °C, resulting in 32 treatment combinations in total.

For each treatment combination, 500 g of equilibrated wheat was treated in a 1 L glass Duran bottle by adding the formulation on top of the grain and then rolling the jar using a bottle roller for 3 min to ensure even distribution of the treatment. The wheat was then subdivided into ten 125 mL glass jars each containing 50 g of grain. Fifteen *S. granarius* adults were added to each jar. To prevent the insects from escaping, the tops of the jars were covered with a square of gauze which was retained in position with an elastic band.

Jars were arranged in one of two humidity controlled incubators set to constant darkness and 65% ± 5% RH. One incubator was set to 15 ± 2 °C and the other to 25 ± 2 °C. At day 14, ten of the jars from each temperature × kaolin × fungal rate combination were randomly selected and the insects assessed as detailed in Section 2.4. The remaining jars were checked at day 28.

### 2.8. Statistical Analysis

For bioassay 1, the mortality data were found to deviate significantly from a normal distribution even after data transformation. As a result, the proportions data for each time point were analysed with a Kruskal-Wallis test followed by pairwise comparisons with a two-way Dunn’s procedure. Analyses were performed using XLSTAT (version 2014.1.01.; Addinsoft, Paris, France, 2014).

For bioassay 2 and 3, the mortality data were found to deviate significantly from a normal distribution, even after data transformations. For each bioassay, logistic regression (probit) was used to model the impact of fungal rate on mortality for each rate of kaolin that was tested. The dose variable was converted to a logarithmic scale before analysis. Each regression profile was then used to find the concentration of spores required to achieve the LC50 at each level of kaolin tested. The LC50 was calculated for doses that adequately fit the probit model and assessed using a χ^2^ goodness-of-fit test (*p* > 0.1 indicates a good fit). The analyses were done using the “Dose” add-in for XLSTAT (version 2014.1.01).

For bioassay 4, the *S. granarius* mortality data were found to deviate significantly from a normal distribution even after data transformations. As a result, the proportions data were used to construct a fully factorial Generalised Linear Model (GLM). An ANOVA was applied to the results of the GLM to look for significant effects. Due to overdispersal of residuals, quasibinomial errors were used. Analysis was carried out using R software (version 3.1.0.; The R Foundation for Statistical Computing, Vienna, Austria, 2014). As for bioassay 2 and 3, logistic regression (probit) was used to model the impact of fungal rate on mortality with or without kaolin, at each temperature, using the “Dose” add-in for XLSTAT (version 2014.1.01.).

## 3. Results

### 3.1. Bioassay 1: Initial Testing of O. surinamensis

**Figure 1:**
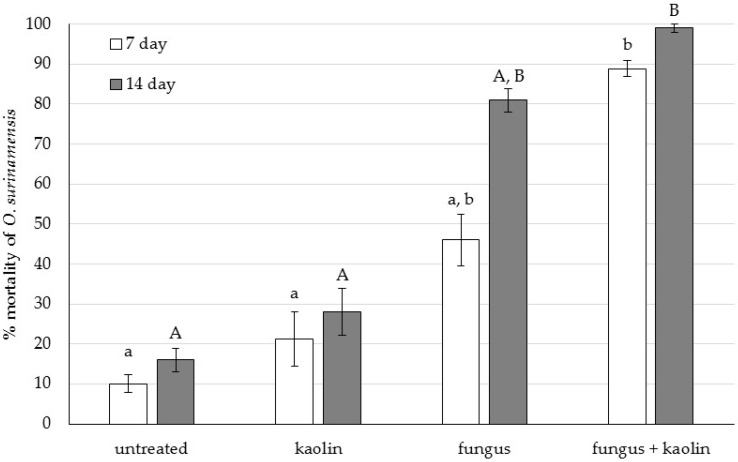
Bioassay 1: Percentage mortality of *Oryzaephilus surinamensis* adults exposed to *Beauveria bassiana* IMI389521 at 1 × 10^10^ conidia per kg of wheat, kaolin at 1.74 g per kg of wheat, and a combination of the two, after 7 and 14 days of exposure (Mean ± SE). Different letters between groups within time points denote statistical difference among treatments (*p* ≤ 0.004).

The effect of treatment was significant for both the day 7 and day 14 mortality data (*p* = 0.001 for both). After 7 d, most of the beetles in the combined treatment were already dead (>88%) (Figure 1) with significantly higher mortality compared to the untreated control and kaolin-only treatments (*p* < 0.001 and *p* = 0.004 respectively), where mortality did not exceed 22%. At day 14, mortality reached 99% in the combined treatment, and was again significantly different than that of the untreated control and kaolin-only groups, (*p* < 0.001 and *p* = 0.003 respectively), where mortality did not exceed 28%. Although mortality in the fungus only treatment (day 7: 46% and day 14: 81%) was less than in the combined treatment, the difference between these groups was not significant (*p* = 0.139 and *p* = 0.174, respectively); possibly owing to the fact that the data were analysed by non-parametric methods and the low number of replications (*n* = 5).

### 3.2. Bioassay 2: Dose Response of O. surinamensis

**Figure 2:**
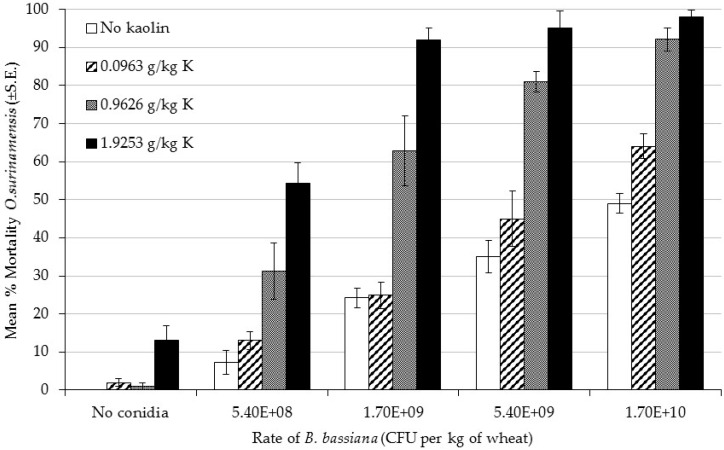
Bioassay 2: Percentage mortality of *Oryzaephilus surinamensis* adults exposed to different rates of *Beauveria bassiana* IMI389521 and different rates of kaolin after 14 days of exposure (Mean ± SE).

The treatments with the most kaolin (1.9253 g per kg of wheat) consistently achieved the highest level of mortality (>90%) when combined with fungus at a rate of at least 1.7 × 10^9^ CFU per kg of wheat or more (Figure 2). The fungus alone never killed more than 50% of the insects, even at the highest rate (1.7 × 10^10^ CFU per kg of wheat). Kaolin alone may have exerted an effect at a rate of 1.9253 g per kg of wheat with mortality at 13% compared to 0% in the untreated control, but this was not tested statistically. Treatments of the fungus with the lowest level of kaolin (0.0963 g per kg of wheat) exhibited similar levels of mortality to the fungus alone. However, when the kaolin was combined with the fungus at 0.9626 or 1.9253 g per kg of wheat, the level of mortality compared to fungus alone was more than doubled in most treatment groups. At the lowest rate of fungus, 5.4 × 10^8^ CFU per kg of wheat, the increase when combined with the highest rate of kaolin was more than seven-fold.

Logistic regression was carried out on the mortality data for each rate of kaolin and was found to adequately fit the probit model (Table 2). The LC50 for fungal rate decreased as the rate of kaolin increased. The LC50 was >12 times lower when kaolin was included at a rate of 0.9629 g per kg of wheat and >44 times lower when it was included at the highest rate of 1.9253 g per kg of wheat.

### 3.3. Bioassay 3: Dose Response of T. confusum Adults and Larvae

**Figure 3:**
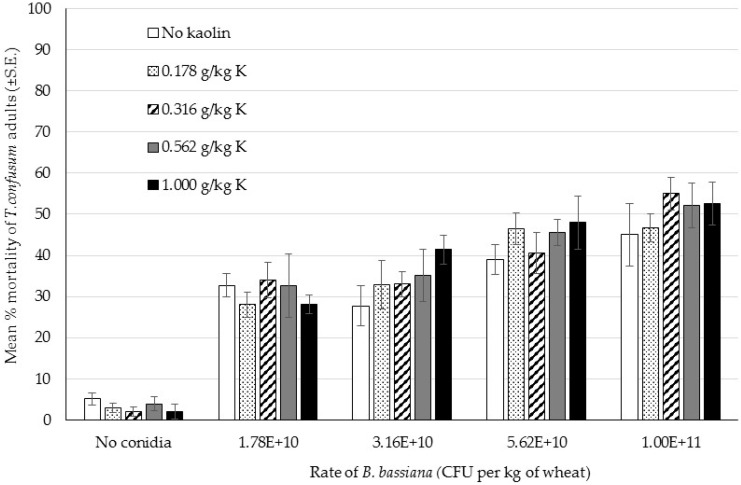
Bioassay 3: Percentage mortality of *Tribolium confusum* adults exposed to different rates of *Beauveria bassiana* IMI389521, and different rates of kaolin, after 14 days of exposure (Mean ± SE).

Mortality did not exceed 55% in any of the treatment groups and above 27% in all groups which contained fungus (Figure 3). In the treatments with no fungus mortality did not exceed 5.2%, even at the highest level of kaolin (1 g per kg of wheat). Kaolin appeared to have very little effect, if any, on mortality, even when combined with fungus. The fungal isolate seemed to exert some efficacy as mortality was higher than in the untreated groups but there was no clear dose response at the range tested (Table 3). The data was a poor fit for the probit model in each case. The LC50 for fungal rate was found to decrease as the rate of kaolin increased but there was little difference between the top and bottom rate with overlapping 95% confidence interval (CI). Neither kaolin rate nor spore rate in the range tested made a large difference to the level of mortality. 

No statistical analysis was carried out on the larval mortality data since adult emergence was zero, or close to zero (0.2% ± 0.18% at 1.78 × 10^10^ with 0.562 g kaolin per kg wheat), in all the treatment groups that contained fungus. In contrast, the adult emergence from the treatment groups that contained no fungus were 53% (no kaolin) and 47% (kaolin at 0.562 g per kg wheat). From this data it is not clear whether kaolin has any effect, either on its own or combined with fungus. However, the data does clearly indicate that the fungus is effective at preventing adult emergence when the larvae are treated with ≥1.78 × 10^10^ CFU per kg wheat.

### 3.4. Bioassay 4: Dose Response of S. granarius and the Effect of Temperature

**Figure 4:**
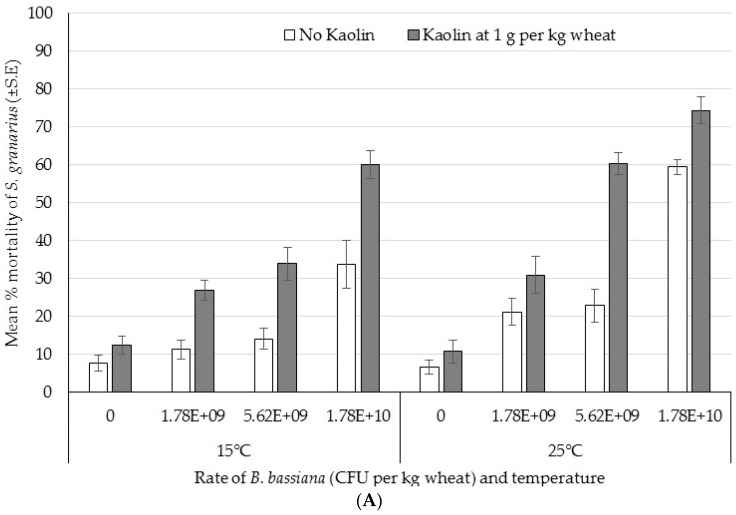

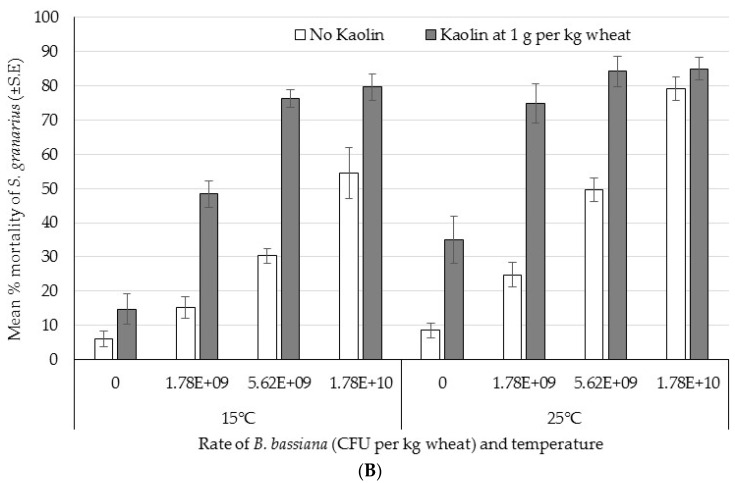
Bioassay 4: Percentage mortality of *Sitophilus granarius* exposed to different rates of *Beauveria bassiana* IMI389521 in colony forming units (CFU) per kg of wheat, with and without kaolin at 1 g per kg of wheat, at 15 or 25 °C after (**A**) 14 days and (**B**) 28 days of exposure (Mean ± SE).

The data clearly indicate that at each time point the level of mortality increased with increasing concentration of *B. bassiana* (Figure 4), and that for each rate of fungus the mortality was increased in the presence of kaolin. The concentration of *B. bassiana* (*F*_(3,312)_ = 212.46, *p* < 0.0001) and the presence of kaolin (*F*_(1,311)_ = 251.68, *p* < 0.0001) were found to be significant effects, as was the effect of temperature (*F*_(1,316)_ = 61.53, *p* < 0.0001) and exposure period (*F*_(1,315)_ = 121.35, *p* < 0.0001). Moreover, significant interactions were found between time and kaolin (*F*_(1,302)_ = 11.93, *p* = 0.0006), fungus and kaolin (*F*_(3,299)_ = 3.77, *p* = 0.011), and time × fungus × kaolin (*F*_(3,293)_ = 2.77, *p* = 0.042). In addition to the mortality increasing with fungal rate and kaolin inclusion, mortality was significantly higher at the second time point. Mortality was also significantly higher at 25 °C than at 15 °C. The significant interaction between time, fungal rate, and kaolin indicates that the degree of synergy between kaolin and fungus was dependent on time. There were bigger differences between formulations with and without kaolin at the second time point than at the first.

Not all of the data were a good fit for the probit model (Table 4), particularly if kaolin had been included in the treatment. At 14 days, including kaolin lowered the LC50 at both temperatures. At 28 days and 15 °C, the LC50 was also lowered when kaolin was included but no LC50 was calculated for the 25 °C data since all dose responses exceeded 50% mortality when kaolin was included. The LC50 at both time points was lower at 25 °C than at 15 °C.

## 4. Discussion

Our studies clearly demonstrate that the addition of even relatively low doses of kaolin clay powder can enhance the efficacy of *B. bassiana* against some adult stored grain beetle pests. Synergistic effects were demonstrated for both *O. surinamensis* and *S. granarius*, though not *T. confusum*. Despite the low susceptibility of *T. confusum* adults, the larvae were highly susceptible to the fungus with close to zero emergence of adult *T. confusum* when larvae were treated with a range of fungal rates, with or without the kaolin. In none of the assays was kaolin by itself effective, which is consistent with information from published literature [10] that as a standalone product, efficacy is only observed at rates ≥5 g per kg of wheat, and the highest rate tested in the present study was 1.9253 g per kg of wheat. Synergistic effects are observed when the effect of two actives taken together is greater than the sum of their separate effect at the same doses. When *O. surinamensis* were treated with a range of fungal and kaolin rates, the mortality in the combined treatment was greater than the sum of the mortality in the corresponding kaolin alone and fungus alone treatment groups when kaolin was ≥0.96 g per kg of wheat. Similarly, in most treatment groups, the mortality of *S. granarius* was greater in the combined formulation (kaolin at 1 g per kg of wheat) than the sum of the mortality caused by kaolin alone or the corresponding fungal rate applied alone. The results indicate synergistic rather than additive effects for these two insect species and are in accordance with results reported by Lord (2001) [29], Akbar et al. (2004) [30], and Sabbour et al. (2012) [6] when the inert dust DE was combined with entomopathogenic fungal spores for control of stored grain beetles and moths. However, as far as we are aware, the current study represents the first demonstration that such a synergistic effect can occur when kaolin is combined with an entomopathogenic fungus.

It is not clear why inert dusts may synergise the effect of entomopathogenic fungi. Akbar et al. (2004) [30] found that the presence of DE increased the conidial attachment of *B. bassiana* on the cuticle of *T. castaneum* larvae, but Lord (2001) [29] reported no significant increase in the case of *R. dominica* larvae. Samodra & Ibraham [32] observed that the waxy layer of the insect’s integument was abraded and removed by the kaolin, which had allowed greater conidial attachment and fungal penetration through the insect exoskeleton. The increased water loss through the insect cuticle as a result of abrasion and absorption of cuticular waxes by inert dusts may result in favourable conditions for spore germination or increase the stress levels of the insect making them more susceptible to fungal infection. A combination of factors may result in their suitability as co-formulants.

The degree of synergism may be highly dependent on temperature and relative humidity. At higher temperatures, water loss is increased and insects are more mobile and so take up more particles on their cuticles [6,15]. Inert dusts are, therefore, generally more effective at higher temperatures. However, there is a threshold at which the activity of the fungus begins to decline. Vassilakos et al. (2006) [36] found that *B. bassiana* (Naturalis^®^ SC, Troy Biosciences, Phoenix, AZ, USA) was more effective against *R. dominica* and *S. oryzae* at 26 °C than at 30 °C. Athanassiou and Steenberg (2007) [25] reported that the efficacy of *B. bassiana* decreased when temperature increased from 25 to 30 °C. The optimum temperature for *B. bassiana* conidial germination and vegetative growth is reported as around 25 °C [37,38]. In the present study, temperature was found to be a significant factor affecting mortality of *S. granarius*, with greater mortality at the higher temperature of 25 °C. This is to be expected as fungal germination is increased with increasing temperature (up to a threshold) and, like DE, the effect of kaolin may be increased at higher temperatures. There was no significant interaction between temperature and the presence or absence of kaolin; the degree of synergism was not greater at 25 °C than at 15 °C. Alterations in the relative humidity were not investigated in the present study, but there may be complex interactions since water stress would increase under lower relative humidity. Inert dusts are more efficacious at lower moisture levels, however, it is not clear how this relationship may change if combined with an entomopathogenic fungus. The effectiveness of *B. bassiana* under different relative humidity varies widely with some studies indicating better efficacy at reduced moisture levels [39,40] and others reporting that the fungus may be less active in drier conditions [41,42]. In the present study, the efficacy with the fungus alone was actually greater at 1 × 10^10^ conidia per kg of wheat in bioassay 1, with around 80% mortality, than at the higher concentration of 1.7 × 10^10^ CFU per kg of wheat in bioassay 2 where mortality was <50%. Although the units of concentration are different, there are usually fewer CFU than conidia per kg of wheat since conidia per kg is the total number of spores present and CFU is the total viable spores present. The main difference between the two studies was that the relative humidity was uncontrolled and consequently lower in bioassay 1, supporting the findings by Lord [39,40] that activity of *B. bassiana*, at least in the case of this isolate, may be better at reduced moisture levels.

The degree of synergism may also depend on the concentrations of each active. Vassilakos et al. (2006) [36] found some additive and some negative effects between *B. bassiana* and the DE product SilicoSec^®^ (Biofa GmbH, Münsingen, Germany) against adults of *S. oryzae* and *R. dominica*, which appeared to be concentration-dependent. At the lowest fungal rates, the addition of DE did not increase the fungal efficacy, and in some cases caused a detrimental effect, and at the highest fungal rates, an additive effect was more often recorded. In the present study, the lowest kaolin rate of 0.0963 g per kg of wheat had neither an additive nor detrimental impact on the mortality caused by the fungus against *O. surinamensis*; both higher rates of kaolin had synergistic effects. In the *Sitophilus* bioassay, no detrimental effects of including kaolin were observed, all effects were either additive or synergistic, and these effects were observed at both temperatures and time points.

The susceptibility of the insects to the fungus and the degree of synergism between kaolin and the fungus is clearly dependent on the species and life stage tested. In the present study, in terms of adult insects, we found *O. surinamensis* to be the most susceptible, followed by *S. granarius*, and then *T. confusum* was the most resistant. This order of susceptibility is in agreement with previous work on this isolate [33]. Resistance of adult *Tribolium* sp. to entomopathogenic fungal infection has been previously reported [30,43,44]. Wakefield (2006) [33] demonstrated, using scanning electron microscopy, that quantitative and qualitative differences in adherence and germination of fungal conidia could be observed between a susceptible species, *O. surinamensis*, and the resistant *T. confusum*. At each of the post-treatment periods, *O. surinamensis* had a greater number of fungal conidia adhering to the cuticle, and the greater adherence appeared to be related to the greater number of setae, particularly on the ventral abdomen, of *O. surinamensis* compared to *T. confusum*. Lord (2007c) [45] showed that *T. castaneum* was more susceptible under desiccation stress. Desiccation stress may be achieved by including an inert dust such as kaolin. However, in the present study, against *T. confusum* adults, the kaolin had no synergistic effect on the fungus at the levels tested, and it was not possible to determine any effects on the larvae since all rates tested resulted in close to 0% emergence of adult beetles. As a result of this, no LC50 was calculated for larvae. Lower rates were not tested on larvae. At day 14 post treatment, an acceptable level of efficacy (>70%) against *S. granarius* was only achieved at the highest rate of 1.78 × 10^10^ CFU per kg of wheat with kaolin at 1 g per kg of wheat, although lower rates were effective on *O. surinamensis*. As the formulation must contain a dose that will be efficacious against all the target species, a rate of 1.78 × 10^10^ CFU per kg of wheat with kaolin at around 1 g per kg of wheat would ensure acceptable efficacy against adult *O. surinamensis*, *S. granarius* and the larval stage of *T. confusum*.

It has previously been observed that *Tribolium* sp. larvae are more susceptible to entomopathogenic fungi than the adult life stage [26,30], an effect which has been shown to synergise with the presence of DE [30]. Kavallieratos et al. (2006) [27] recovered very few progeny of *T. confusum* in wheat treated with *M. anisopliae* and no progeny production was recorded when DE was included. *Tribolium* sp. larvae feed and develop in the external part of the kernels, thus, the chance of encountering fungal conidia is increased compared to that of the adults. In terms of susceptibility to inert dusts, like the fungi, the larvae are much more susceptible to DE than the adults [18,46]. In order to better understand the relationship between kaolin and *B. bassiana* IMI389521 and the potential for any synergy, lower rates of fungus would need to be tested.

It has been demonstrated that the inert dust kaolin can have synergistic effects when combined with an entomopathogenic fungus and results in much higher levels of efficacy against stored grain beetles than would otherwise be possible with the isolate alone. This has been previously demonstrated for an alternative mineral earth powder, DE. Kaolin may convey certain advantages over the use of DE since it is softer and may be less likely to negatively impact processing machinery. While the kaolin demonstrated little effectiveness on *T. confusum* adults, the isolate tested, IMI389521, already demonstrated high levels of efficacy on the larvae, which may in fact be a better target. By targeting the larvae rather than the adults there is a reduced possibility that adults will emerge, mate, and lay eggs before the fungal infection has been able to take effect. It would be worthwhile to screen the isolate and co-formulant kaolin on the life stages of other grain beetles, and, perhaps, other important grain pests such as moths and psocids.

## 5. Conclusions

Kaolin is a widely available and relatively inexpensive material. It is already known as a non-synthetic control product with insect repellent properties, and it is currently used in particle film technology for protection of a wide variety of agricultural crops [47,48]. This research has now demonstrated the potential for kaolin as a co-formulant for entomopathogenic fungal control, in this case, for use in stored grain. By synergizing the effect of the fungus, the co-formulant kaolin may overcome the lower levels of efficacy that are perceived as a barrier to the widespread adoption of microbial control agents in storage systems.

## Figures and Tables

**Table 1:** Details of the application rates and quantities of formulation components used in the *Sitophilus granarius* bioassay (bioassay 4).

Formulation Applied to Wheat (g per kg)	Rate of Conidia Applied to Wheat (CFU per kg)	Amount in Formulation (g per kg)			
		Fungal Conidia	Kaolin	Entostat	Sipernat d17
0.0507	1.78E+9	350	0	645	5
0.1606	5.62E+10	350	0	645	5
0.5086	1.78E+10	350	0	645	5
1.0509	1.78E+9	16.9	951.6	26.5	5
1.1605	5.62E+10	48.4	861.6	84.9	5
1.5086	1.78E+10	118	662.9	214.1	5

**Table 2:** Results of logistic regression on *Oryzaephilus surinamensis* mortality data for each rate of kaolin in dose response bioassay 2.

Rate of Kaolin (g per kg of Wheat)	LC50 (CFU per kg of Wheat)	Lower Bound 95%	Upper Bound 95%	R^2^	χ^2^	*p*-Value
0	1.582E+10	1.008E+10	3.230E+10	0.099	47.541	<0.0001
0.0963	7.47E+09	5.43E+09	1.13E+10	0.129	67.925	<0.0001
0.9626	1.228E+09	8.752E+08	1.606E+09	0.202	96.657	<0.0001
1.9253	3.586E+08	2.045E+08	5.174E+08	0.230	78.411	<0.0001

**Table 3:** Results of logistic regression on *Tribolium confusum* adult mortality data for each rate of kaolin in dose response bioassay 3.

Rate of Kaolin (g per kg of Wheat)	LC50 (CFU per kg of Wheat)	Lower Bound 95%	Upper Bound 95%	R^2^	χ^2^	*p*-Value
0	1.99E+11	8.86E+10	3.48E+15	0.013	7.008	0.008
0.178	1.07E+11	6.72E+10	5.04E+11	0.020	10.885	0.001
0.316	8.97E+10	5.83E+10	3.28E+11	0.020	10.612	0.001
0.562	8.60E+10	5.65E+10	2.92E+11	0.021	11.352	0.001
1.000	7.23E+10	5.07E+10	1.58E+11	0.025	13.459	>0.001

**Table 4:** Results of logistic regression on *Sitophilus granarius* adult mortality data for each treatment group (time × temp. × kaolin) in dose response bioassay 4.

Days after Treatment	Temperature (°C)	Kaolin	LC50 (CFU per kg of Wheat)	Lower Bound 95%	Upper Bound 95%	R^2^	χ^2^	*p*-Value
14	15	No	4.21E+10	2.48E+10	2.54E+11	0.180	33.154	<0.0001
		Yes	1.12E+10	8.22E+09	1.73E+10	0.081	40.373	<0.0001
	25	No	1.44E+10	1.11E+10	2.08E+10	0.117	54.743	<0.0001
		Yes	4.28E+09	3.24E+09	5.45E+09	0.087	62.332	<0.0001
28	15	No	1.49E+10	1.13E+10	2.22E+10	0.123	54.862	<0.0001
		Yes	1.51E+09	7.09E+08	2.32E+09	0.036	33.985	<0.0001
	25	No	5.49E+09	4.46E+09	6.62E+09	0.149	103.483	<0.0001
		Yes	No analysis possible, all responses >50%					
